# Pancreatic hamartoma: a case report and literature review

**DOI:** 10.1186/s12876-016-0419-2

**Published:** 2016-01-14

**Authors:** Daisuke Matsushita, Hiroshi Kurahara, Yuko Mataki, Kosei Maemura, Michiyo Higashi, Satoshi Iino, Masahiko Sakoda, Hiroyuki Shinchi, Shinichi Ueno, Shoji Natsugoe

**Affiliations:** Department of Digestive Surgery and Breast and Thyroid Surgery, Graduate School of Medical and Dental Sciences, Kagoshima University, 8-35-1 Sakuragaoka, Kagoshima, 890-8520 Japan; Department of Human pathology, Field of Oncology, Graduate School of Medical and Dental Sciences, Kagoshima University, Kagoshima, Japan; Faculty of Medical School of Health Sciencesy, Graduate School of Medical and Dental Sciences, Kagoshima University, Kagoshima, Japan

**Keywords:** Pancreas, Hamartoma, Cystic lesion, Pseudotumor, Cytokeratin, S-100

## Abstract

**Background:**

Pancreatic hamartoma is an extremely rare benign disease of the pancreas. Only 30 cases have been reported to date.

**Case presentation:**

A 68-year-old man presented with an asymptomatic solid and multi-cystic lesion in the uncus of the pancreas, incidentally detected on abdominal enhanced computed tomography. The tumor was found to be a well-demarcated solid and multi-cystic lesion without any enhancement, measuring 4 cm in diameter. After 28 months of follow-up, the tumor enlarged. At 31 months after initial diagnosis, the patient underwent surgical resection because it was difficult to clinically determine whether the tumor was malignant or not. Macroscopically, the solid tumor consisted of yellow adipose tissue with a smooth thin capsule confined to the pancreatic uncus. The inner structure of the tumor consisted of multiple cysts with a white nodule between the cysts. Histologically, the solid part and the multi-cystic portion consisted of mature adipose tissue and colonization of dilated pancreatic ducts with mild fibrosis, respectively. Immunohistochemical findings revealed cytokeratin 7 and 19 positive staining in the epithelial cells of the ducts. Adipose tissue showed positive staining for S-100 protein and there were only a few MIB-1 positive cells. The tumor was then diagnosed as a pancreatic hamartoma.

**Conclusion:**

Beside on the above findings, we suggest that the term “well-demarcated solid and cystic lesion with chronological morphological changes” could be a clinical keyword to describe pancreatic hamartomas.

**Electronic supplementary material:**

The online version of this article (doi:10.1186/s12876-016-0419-2) contains supplementary material, which is available to authorized users.

## Background

Pancreatic tumor-like cystic lesions are significantly less common than solid lesions, and they account for less than 1 % of all pancreatic tumors [1]. Cystic tumors of the pancreas are categorized as pseudocysts and true lined cysts (e.g., intraductal papillary mucinous neoplasm [IPMN], mucinous cystic neoplasm [MCN], and serous cystic neoplasm [SCN]). Rare pancreatic cysts such as squamous-lined cysts (e.g., lymphoepithelial cyst, epidermoid cyst, dermoid cyst, and squamoid cyst) and solid-pseudopapillary neoplasms (SPNs) have also been reported. In addition, extremely rare non-neoplastic pancreatic tumors such as hamartomas have been reported. The differential clinical diagnosis of these pancreatic benign tumors from malignant disease using abdominal ultrasound (AUS), computed tomography (CT), magnetic resonance imaging (MRI), endoscopic retrograde cholangiopancreatography (ERCP), and endoscopic ultrasound (EUS) remains difficult despite advances in diagnostic imaging equipment [2]. Therefore, histological diagnosis with surgical resection is often needed to diagnose these rare tumors. Here, we report a case of pancreatic hamartoma and review the English literature on this extremely rare pancreatic tumor.

## Case presentation

**Fig. 1:**
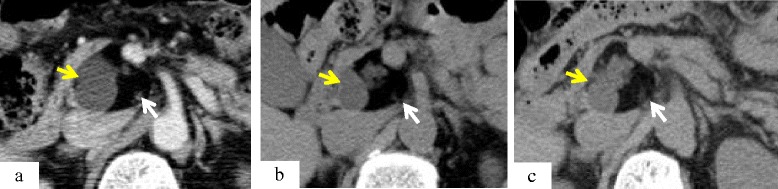
Chronological changes seen on CT. **a**: First examination. The hypo-enhanced mass was 4.2 × 3.9 cm in size with solid and cystic lesions located in the uncus of the pancreas. **b**: At 21 months after first examination. The tumor shown is 3.9 × 3.6 cm in size. The cystic lesion (*yellow arrow*) had become smaller and the solid lesion (*white arrow*) had become larger. **c**: At 28 months after first examination. The tumor was 4.2 × 3.3 cm in size. The cystic lesion changed and displayed irregular margins

**Fig. 2:**
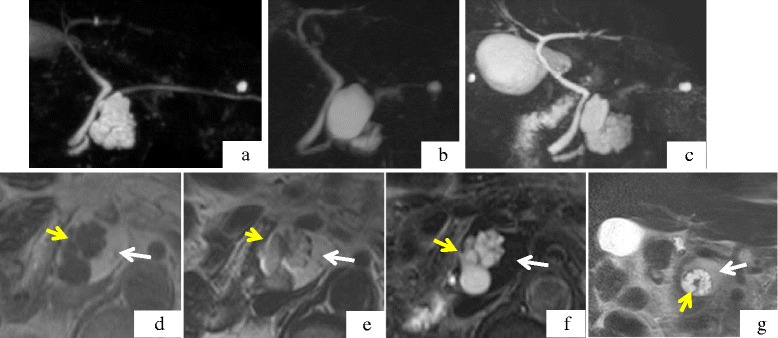
**a**, **b**, **c**. Chronological changes seen on MRCP. **a**: At the first examination. The tumor was observed as multiple cystic lesions. **b**: At 21 months after first examination. **c**: At 28 months after first examination. Figure 2-**d**, **e**, **f**, **g**. MRI appearance at 28 months after first examination. **d**: T1WI. A multi-cystic lesion with low intensity was surrounded by a mass with iso-intensity. **e**: T2WI. A multi-cystic lesion displays low- to iso-intensity. The surrounding tissue shows iso- to high intensity. **f**: T2-FAT-SAT. A multi-cystic high intensity lesion is shown. The surrounding tissue shows complete fat suppression. **g**: T2WI (coronal image). A ringed multi-cystic lesion with high intensity is surrounded by a smooth superficial mass. A small nodule is seen inside of the cyst ring

A 68-year-old man presented with an asymptomatic solid and multi-cystic mass in the uncus of the pancreas, incidentally detected on abdominal enhanced CT during a health examination. The patient had no relevant medical history, including chronic pancreatitis. The tumor was demonstrated to be a well demarcated solid and cystic lesion, measuring 4.2 × 3.9 cm in diameter by enhanced CT (Fig. 1a). The tumor consisted of a cystic lesion with iso- to low-density surrounded by an extremely low-density area similar in density to adipose tissue. MRI and magnetic resonance cholangiopancreatography (MRCP) showed multilocular cysts ranging in size from one to several millimeters in the pancreas uncus. The main pancreatic duct (MPD) showed no dilation and the evidence of communications between the tumor and MPD was not found (Fig. 2a). The morphological feature of the tumor was completely different from pancreatic adenocarcinoma and the tumor was initially diagnosed benign tumor such as lipoma, dermoid cyst or the other rare benign tumor (Table 1). During the first enhanced CT examination, the patient developed an allergic reaction to contrast medium so that the patient was followed-up regularly with plain CT and MRI.

**Table 1:** Differential diagnosis of the rare cystic lesion of the pancreas

	Age	Gender	Region	Morphology	Contents of the cyst	Histological feature
Lymphoepithelial cyst 1)20)21)	50s-60s	Male		Uni- or multilocular	Serous to cheesy/caseous-appearing depending on the degree of keratin formation.	Lined by well-differentiated stratified squamous epithelium. Surrounding dense lymphoid tissue.
				Cyst wall and trabeculae are usually thin.		
Epidermoid cyst 1)22)	20s-30s	Female	Tail	Uni- or multilocular	Serous to cheesy/casseous-appearing depending on the degree of keratin formation.	Lined by attenuated squamous cells. Exist with accessory spleens.
					High levels of CA 19-9 and/or CEA in the serum and in the cystic fluid.	
Dermoid cyst 1)23)24)	20s-30s	Unknown		Similar to the teratoma	Cheesy or caseous, with keratinaceous and sebaceous secretions.	Skin appendages and sebaceous glands, hair follicles, etc.
Squamoid cyst 1)25)26)	Unknown	Unknown		Unilocular	Acidophilic acinar	Cystically dilated ducts lined by a squamous/transitional epithelium.
						Basal are positive for p63.
						Superficial cells are positive for MUC1, MUC6 and involucrin.
Serous cystadenoma 1)27)	60s	M:F=1: 3	Body and tail	Multi-cystic large mass (mean size: 6 cm)	Serous fluid	Cuboidal glycogen-rich epithelial cells positive for GLUT-1.
					Sponge-like appearance	Clear cytoplasm and well-defined cytoplasmic borders. Small, round nuclei with dense homogeneous chromatin.
						Von Hippel-Lindau gene is detected in 40% cases.
Lipoma 28)29)30)	Unknown	Unknown		Hypodensity (-30 to -120 HU) and homogeneity in enhanced CT	Mature adipose tissue, capsuled by thin collagen layer	No evidence of typical pancreatic tissue.
Hamartoma 1),3)-19)	50s- 60s	M:F=1.4:1	Head	Solid and cystic mass	Mature acini, ducts with architectural disarrangement surrounded by stromal fibrosis.	C34, CD117 or bcr-2 expression for the stromal fibrosis.
					Lack or decrease of islet cells.	S-100 protein expression for the ductal component.

CA19-9; Carbohydrate antigen 19-9, CEA: Carcinoembryonic antigen, GLUT-1: Glucose transporter -1

The patient visited for re-examination 21 months after the initial visit because he had no symptoms. The size of the pancreatic tumor had become smaller, measuring 3.9 cm in maximum diameter, and the internal structure showed morphological changes with smaller cysts and a larger solid lesion by plain CT (Fig. 1b) and MRI/MRCP (Fig. 2b). The tumor displayed a honeycomb-like appearance by EUS. At 28 months after the initial visit, the tumor showed an increase in size by plain CT. MRCP showed an increase in the size and number of the cysts, with no infiltration into the pancreatic ducts (Figs. 1c and 2c). The multi-cystic lesion displayed low intensity on T1-weighted imaging (T1WI), iso-intensity on T2-weighted imaging (T2WI) and high intensity on T2-fat saturation (FAT-SAT) by MRI. The solid lesion surrounding the multi cystic lesion displayed iso-intensity on T1WI, iso- to high intensity on T2WI and low intensity with complete fat-tissue suppression using T2-FATSAT conditions (Fig. 2d-f). These findings demonstrated that the cystic lesion consisted with pancreatic juice and the solid lesion was a fat tissue.

On coronal T2WI imaging, ringed-multilocular cysts with high intensity were surrounded by a smooth superficial mass, and a small nodule was observed inside of the cyst ring (Fig. 2g). These images suggested the possibility of IPMN malignant transformation. At 31 months after the initial diagnosis, the patient underwent surgical resection. There were no symptoms or abnormal levels of tumor markers (carcinoembryonic antigen [CEA], carbohydrate antigen 19-9 [CA19-9]) during follow-up.

### Operative findings

**Fig. 3:**
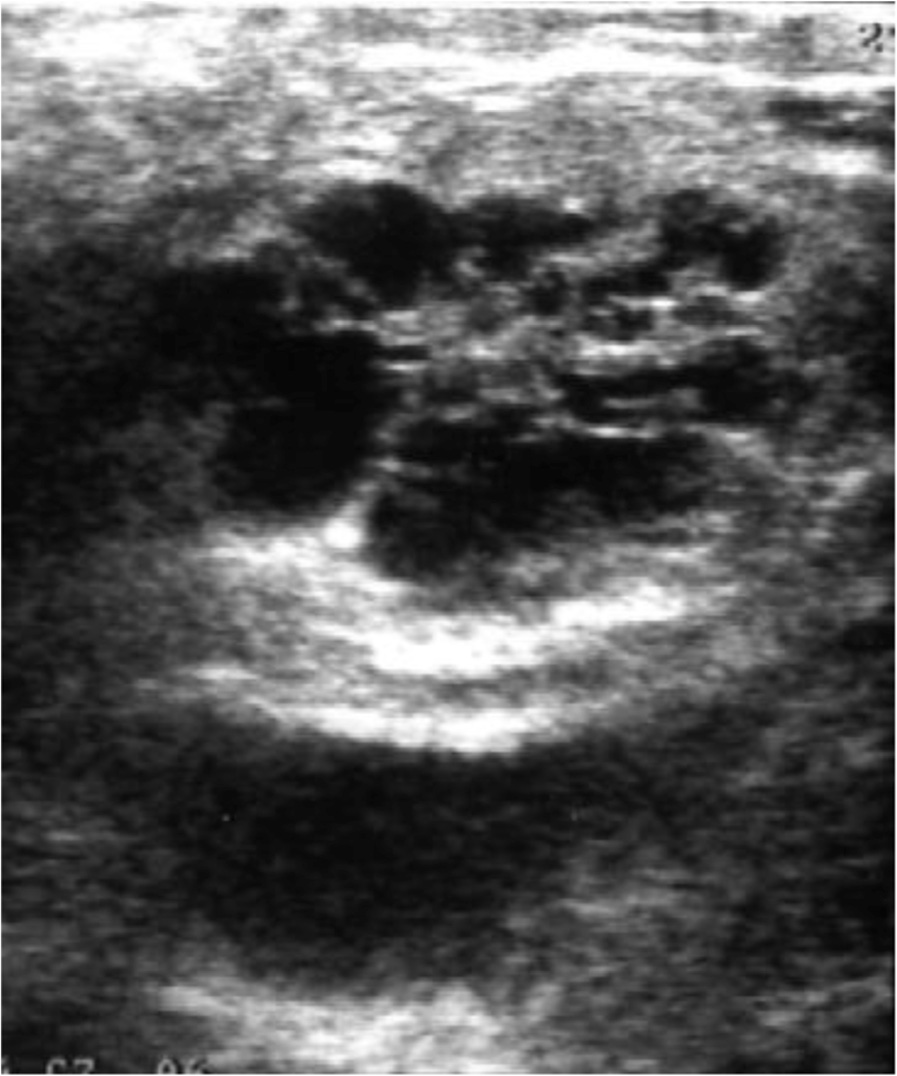
Intraoperative ultrasound examination. A well-demarcated honeycomb-like cystic lesion in the pancreatic uncus was found. There was no evidence of venous or ductal invasion

A soft and elastic tumor was confirmed in the uncus of the pancreas. The tumor was not exposed and there were no inflammatory changes around the pancreas. Intraoperative ultrasound showed a well demarcated honeycomb-like cystic lesion in the pancreatic uncus, and there was no evidence of venous or ductal invasion (Fig. 3). Pylorus preserving pancreaticoduodenectomy was performed. The operation was completed without any complications.

### Macroscopic findings

**Fig. 4:**
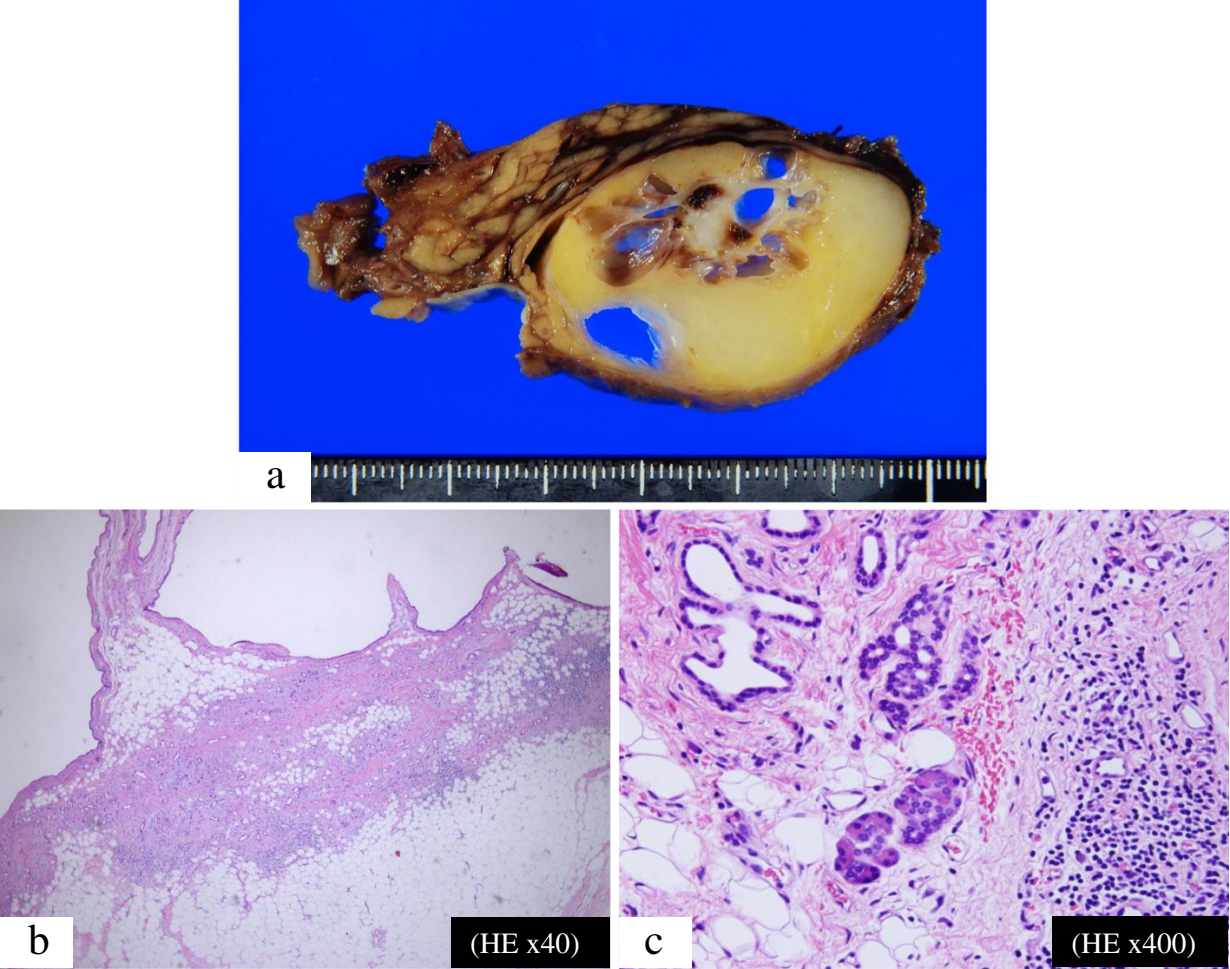
Macroscopic and pathological findings of the pancreatic hamartoma. **a**: Solid and cystic tumor in the pancreatic uncus with a smooth, thin capsule. Cysts were surrounded by yellow adipose tissue and a white nodule was found between the cysts. **b**: The solid lesion filled with mature adipose tissue. There was no evidence of mucinous products in the cysts and no evidence of malignancy in the epithelial cells of the cysts. **c**: The cystic lesion consisted of dilated ducts. Fibrosis and infiltration of monocytes were observed around the cysts. There were a few normal acini in the solid lesion, and the nodule located between the cysts consisted of stromal fibrosis without atypical cells

A solid tumor consisting of yellow adipose tissue with a smooth thin capsule measuring 40 × 40 × 28 mm in size was confirmed in the pancreatic uncus. The inner structure of the tumor consisted of multiple cysts, and a white nodule was found between the cysts (Fig. 4a).

### Hematoxylin-eosin staining (HE)

The solid part of the mass consisted of mature adipose tissue, and the cystic lesion consisted of colonization of the dilated pancreatic ducts with mild fibrosis and infiltration of monocytes in the circumference. There was no evidence of mucinous products in the cysts, and no evidence of malignancy in the epithelial cells of the cysts. A few normal scattered acini were observed in the solid portion of the lesion, and the nodule located between the cysts consisted of stromal fibrosis without atypical cells (Fig. 4b).

### Immunohistochemical staining (IHC)

**Fig. 5:**
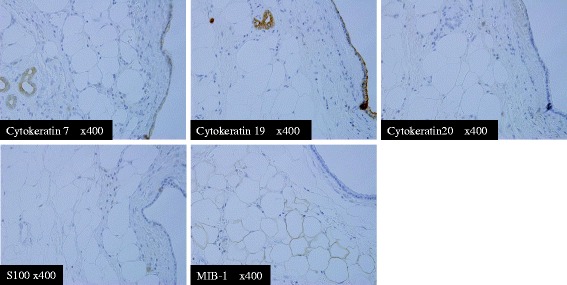
Immunohistochemical stains. The epithelial cells of the ducts expressed cytokeratin 7 and cytokeratin 19, but were negative for cytokeratin 20. The adipose tissue was positive for S-100 protein and mostly negative for MIB-1

The epithelial cells of the ducts expressed cytokeratin 7 and cytokeratin 19, but were negative for cytokeratin 20. The adipose tissue was positive for S-100 protein, and mostly negative for MIB-1 (Ki-67). These results suggest that the tumor was non-proliferative (Fig. 5). Based on these findings, the tumor was determined to be composed of a mixture of differentiated cell types that are normally present in the pancreas. It was therefore regarded as a malformation rather than a neoplasm. Giving these findings, the tumor was diagnosed as a pancreatic hamartoma.

After surgical resection, the patient received follow-up examination with plain CT every three to six months and there was no recurrence for 50 months.

## Discussion

Pancreatic hamartoma is an extremely rare benign disease of the pancreas. With the improvements in diagnostic imaging equipment over the last decade, it has become increasingly common to discover rare pancreatic tumors such as those listed above. Although these tumors are usually not aggressive, they typically require surgical resection because of the difficulty in prospective clinical diagnosis [2]. Accurate knowledge about these rare pancreatic tumors is therefore necessary. Table 1 shows the clinical features of the rare pancreatic tumors for which differential diagnosis was carried out in the present case [1, 3–31]. The information in Table 1 provides the clinical and histological characteristics of each disease. However, this information alone is not sufficient to be used as decisive diagnostic evidence.

Albrecht [32] first introduced the term “hamartoma” to describe “tumor-like malformations” of the liver, spleen, kidney, and breast that show an abnormal admixture of normal components typical of the organ involved. A hamartoma may be regarded as a malformation rather than a neoplasm. A pancreatic hamartoma is an extremely rare pancreatic tumor, accounting for < 1 % of occurrences of tumor-like cystic lesions of the pancreas [15]. There are only 30 cases of pancreatic hamartoma reported in the English literature to date [3–20], including the first case reported by Anthony et al. in 1977 [3]. All of these reports have described the difficulty in imaging diagnosis, and almost all patients underwent surgical resection because of the possibility of malignant disease.

Table 2 (Additional file 1 shows detailed data) shows the clinical and pathological features of 31 cases of pancreatic hamartoma, including the present case. The median age of the patients was 50.4 years (range, 34 weeks to 78 years), and there were no significant differences in the male-to-female ratio (1.4/1.0). Thirteen of the patients had no clinical symptoms, and the other patients had non-typical symptoms such as abdominal pain and weight loss. Noltenius et al. [33] suggested a relationship between the pancreatic hamartoma-like appearance and chronic pancreatitis in a case study of Wernicke’s encephalopathy with alcoholic pancreatitis. In this study, only 4 cases were complicated by pancreatitis. On the other hand, many authors, including Pauser et al. [10], suggested that hamartoma should be distinguished from pancreatitis because chronic pancreatitis may just mimic hamartoma lesions, while lacking acinar cells.

**Table 2:** A summary of the literature review of pancreatic hamartoma

	Age	Mean (range)	50.5 years (34 weeks - 78 years)
	Sex	M/F	18/12
	Site	Head/body & tail/diffuse	20/8/2
Clinical features	Size	Mean (range)	4.4 cm (0.9 – 19 cm)
	Treatment	PD/other	11/19
	Symptom	+/-	18/12
	Pancreatitis	+/-	4/26
	Acini	+/-	28/0*
	Islets	+/-	10/16*
	Ducts	+/-	29/0*
	Fibrous stroma	+/-	28/2
	Solid/Cystic	Solid/solid and cystic	14/14*
Histopathological features	Solitary/Multiple	Solitary/multiple	22/5*
	Immunostaining	CD34	15 cases
		CD117	9 cases
		S-100	11 cases
		CK 7/8/19	3 cases
		bcr-2	3 cases
		Ki-67	1 cases

*Lack of some cases were not demonstrated in this table

Regarding the morphological features of these cases, 15 cases showed a solid pattern, and 14 cases showed both a solid and cystic pattern (this was unknown in two cases). A solitary tumor was observed in 22 patients, and multiple tumors were found in 6 cases (this was unknown in three cases). All cases showed a well-demarcated line and non-invasive growth. The median size of the main tumor was 4.4 cm (range, 0.9–19 cm). Twenty-one of the reported hamartomas were located in the head, four were located in the body, and 4 in the tail of the pancreas. Two cases were identified as diffuse tumors.

There were only a few patients who could be followed for a long period of time. In the present case, we were able to follow the patient over 2 years, during which we observed chronological morphological changes in the tumor. Sueyoshi et al. [14] reported transformation of the main tumor from multiple large cysts to multiple micro-cysts with solid components in two months. We suggest that this type of chronological morphological change is likely one of the clinical features of pancreatic hamartoma.

Regarding the pathological findings, almost all cases demonstrated disarranged acinar and ductal cells embedded in the fibrous stroma. The solid lesion consisted of fibrous and adipose tissue, and the cystic lesion consisted of dilated ducts. The ducts varied in size and were lined by columnar epithelium without atypical cells. The acinar cells were well differentiated without normal lobular structures. In contrast, normal islets of Langerhans were confirmed in only 9 cases. Pauser et al. [9, 10] and Yamaguchi et al. [19] defined the criteria for the diagnosis of pancreatic hamartoma as: (i) forming a well-demarcated mass, (ii) being comprised of mature acini and ducts with distorted architecture, and (iii) lacking discrete islets of Langerhans. When other case reports are taken into consideration, the presence or absence of the islet cells is still controversial.

In immunohistochemical studies, several authors reported that the acinar cells of pancreatic hamartomas were positive for exocrine markers (amylase and trypsin), and that the ductal cells were positive for epithelial markers (CAM5.2, AE1/AE3 and EMA) [7, 9, 11, 17], similar to what is observed in a normal pancreas. In the present case, cytokeratin 7 and cytokeratin 19, two typical epithelial markers, were expressed in the ductal cells. Conversely, expression of cytokeratin 20, a marker of colorectal and bile ductal epithelium, was negative. Some studies have reported negative staining for S-100 protein, α-smooth muscle actin (α-SMA), desmin and bcr-2 in the stroma cells [11, 15, 17]. In contrast, other cases, including the present case, showed positive staining for S-100 protein in the mature adipose tissue and ductal components [12, 19, 20].

Nagata et al. [11] reported that some of the disordered acinar cells, ductal epithelium, and stromal cells expressed Ki-67. In contrast, Kim et al. [15] and the present case demonstrated negative staining for MIB-1. Recently, many authors have reported that stromal spindle cells express CD34 and CD117 [9–11, 15, 17, 19, 20]. CD34 is a myeloid stem-cell marker and is thought to play an important role in maintaining stromal integrity and inhibiting tumor cell migration. CD117 is a transmembrane tyrosine kinase receptor for stem cell factors and is encoded by the proto-oncogene c-kit [10]. As mentioned above, the characteristics of pancreatic hamartoma are still unclear and controversial. This causes preoperative diagnosis to be difficult, and histological diagnosis with surgical resection is often needed. More reports are necessary to clarify the clinicopathological features of pancreatic hamartomas.

## Conclusion

Pancreatic hamartoma is an extremely rare tumor of the pancreas. Recently, this disease has become clearly recognized, the clinical imaging diagnosis remains some of difficult. We suggest that the term “well-demarcated solid and cystic lesion with chronological morphological changes” could be a clinical keyword to describe pancreatic hamartomas.

## Consent

Written informed consent was obtained from the patient for publication of this case report and any accompanying images. A copy of the written consent is available for review by the Editor of this journal.

## Additional file

Additional file 1:
**A literature review of the pancreatic hamartoma.** (DOC 86 kb)

## Abbreviations

α-SMA: α-smooth muscle actin
AUS: abdominal ultrasound
CA19-9: carbohydrate antigen 19-9
CEA: carcinoembryonic antigen
CT: computed tomography
ERCP: endoscopic retrograde cholangiopancreatography
EUS: endoscopic ultrasound
FAT-SAT: T2-fat saturation
HE: Hematoxylin-eosin staining
IHC: immunohistochemical staining
IPMN: Intraductal papillary mucinous neoplasm
MCN: mucinous cystic neoplasm
MPD: main pancreatic duct
MRCP: magnetic resonance cholangiopancreatography
MRI: magnetic resonance imaging
SCN: serous cystic neoplasm
SPNs: solid-pseudopapillary neoplasms
T1WI: T1-weighted imaging
T2WI: T2-weighted imaging

## References

[1] Volkan Adsay AN (2007). Cystic lesions of the pancreas. Mod Pathol.

[2] Raman SP, Hruban RH, Cameron JL, Wolfgang CL, Fishman EK (2012). Pancreatic imaging mimics: part 2, pancreatic neuroendocrine tumors and their mimics. AJR Am J Roentgenol.

[3] Anthony PP, Faber RG, Russell RC (1977). Pseudotumours of the pancreas. Br Med J.

[4] Burt TB, Condon VR, Matlak ME (1983). Fetal pancreatic hamartoma. Pediatr Radiol.

[5] Flaherty MJ, Benjamin DR (1992). Multicystic pancreatic hamartoma: a distinctive lesion with immunohistochemical and ultrastructural study. Hum Pathol.

[6] Izbicki JR, Knoefel WT, Müller-Höcker J, Mandelkow HK (1994). Pancreatic hamartoma: a benign tumor of the pancreas. Am J Gastroenterol.

[7] Wu SS, Vargas HI, French SW (1998). Pancreatic hamartoma with Langerhans cell histiocytosis in a draining lymph node. Histopathology.

[8] McFaul CD, Vitone LJ, Campbell F, Azadeh B, Hughes ML, Garvey CJ (2004). Pancreatic hamartoma. Pancreatology.

[9] Pauser U, Kosmahl M, Kruslin B, Kilmstra DS, Klöppel G (2005). Pancreatic solid and cystic hamartoma in adults: characterization of a new tumorous lesion. Am J Surg Pathol.

[10] Pauser U, da Silva MT, Placke J, Kilmstra DS, Klöppel G (2005). Cellular hamartoma resembling gastrointestinal stroma tumor: a solid tumor of the pancreas expressing c-kit (CD117). Med Pathol.

[11] Nagata S, Yamaguchi K, Inoue T, Yamaguchi H, Ito T, Gibo J (2007). Solid pancreatic hamartoma. Pathol Int.

[12] Durczynski A, Wiszniewski M, Olejniczak W, Polkowski M, Sporny S, Strzelczyk J (2011). Asymptomatic solid pancreatic hamartoma. Arch Med Sci.

[13] Kersting S, Janot MS, Munding J, Suelberg D, Tannapfel A, Chromik AM (2012). Rare solid tumors of the pancreas as differential diagnosis of pancreatic adenocarcinoma. JOP.

[14] Sueyoshi R, Okazaki T, Lane GJ, Arakawa A, Yao T, Yamataka A (2013). Multicystic adenomatoid pancreatic hamartoma in a child: Case report and literature review. Int J Surg Case Rep.

[15] Kim HH, Cho CK, Hur YH, Koh YS, Kim JC, Kim HJ (2012). Pancreatic hamartoma diagnosed after surgical resection. J Korean Surg Soc.

[16] Sampelean D, Adam M, Muntean V, Hanescu B, Domsa I (2009). Pancreatic hamartoma and SAPHO syndrome: a case report. J Gastrointestin Liver Dis.

[17] Kawakami F, Shimizu M, Yamaguchi H, Hara S, Matsumoto I, Ku Y (2012). Multiple solid pancreatic hamartomas: A case report and review of the literature. World J Gastrointest Oncol.

[18] Addeo P, Tudor G, Oussoultzoglou E, Averous G, Bachellier P (2014). Pancreatic hamartoma. Surg.

[19] Yamaguchi H, Aishima S, Oda Y, Mizukami H, Tajiri T, Yamada S (2013). Distinctive histopathological findings of pancreatic hamartomas suggesting their “hamartomatous” nature: a study of 9 cases. Am J Surg Pathol.

[20] Inoue H, Tameda M, Yamada R, Tano S, Kasturahara M, Hamada Y (2014). Pancreatic hamartoma: a rare cause of obstructive jaundice. Endoscopy.

[21] Mandavilli SR, Port J, Ali SZ (1999). Lymphoepithelial cyst (LEC) of the pancreas: cytomorphology and differential diagnosis on fine-needle aspiration (FNA). Diagn Cytopathol.

[22] Tewari N, Rollins K, Wu J, Kaye P, Lobo DN (2014). Lymphoepithelial cyst of the pancreas and elevated cyst fluid carcinoembryonic antigen: a diagnostic challenge. JOP..

[23] Zavras N, Machairas N, Foukas P, Lazaris A, Patapis P, Machairas A (2014). Epidermoid cyst of an intrapancreatic accessory spleen: a case report and literature review. World J Surg Oncol..

[24] Salimi J, Karbakhsh M, Dolatshahi S, Ahmadi SA (2004). Cystic teratoma of the pancreas: a case report. Ann Saudi Med..

[25] Scheele J, Barth TF, Strassburg J, Juchems M, Kornmann M, Henne-Bruns D (2010). Dermoid cyst of the pancreas. Int J Colorectal Dis..

[26] Kurahara H, Shinchi H, Mataki Y, Maeda S, Takao S (2009). A case of squamous cyst of pancreatic ducts. Pancreas..

[27] Milanetto AC, Iaria L, Alaggio R, Pedrazzoli S, Pasquali C (2013). Squamous cyst of pancreatic ducts: a challenging differential diagnosis among benign pancreatic cysts. JOP..

[28] Reid MD, Choi H, Balci S, Akkas G, Adsay V (2014). Serous cystic neoplasms of the pancreas: clinicopathologic and molecular characteristics. Semin Diagn Pathol..

[29] Hois EL, Hibbeln JF, Sclamberg JS (2006). CT appearance of incidental pancreatic lipomas: a case series. Abdom Imaging..

[30] Suzuki R, Irisawa A, Hikichi T, Shibukawa G, Takagi T, Wakatsuki T (2009). Pancreatic lipoma diagnosed using EUS-FNA. A case report. JOP..

[31] Wang H, Li K, Wang J (2011). A large lipoma of the pancreas. ANZ J Surg..

[32] Albrecht E (1904). Uber hamartoma. Verhandlungen der Deutschen Gesellschaft fur Pathologie..

[33] Noltenius H, Colmant HJ (1977). Excessive hyperplasia of the exocrine pancreatic tissue and Wernicke’s encephalopathy (author’s transl). Med Klin.

