# Supplementary material for: Pancreatic hamartoma: a case report and literature review
Source: BMC Gastroenterol. 2016 Jan 14;16:3. doi: 10.1186/s12876-016-0419-2 (PMC4712467; doi:10.1186/s12876-016-0419-2)
Supplement: Additional file 1: — A literature review of the pancreatic hamartoma. (DOC 86 kb) [file 12876_2016_419_MOESM1_ESM.doc]

Additional file 1. A literature review of the pancreatic hamartoma.

| Case | Author (Ref.) | Age | Sex | Site | Size  (cm) | Treatment | Symptom | Pancreatitis | Acini | Islets | Ducts | Fibrous stroma | Solid / Cystic | Solitary / Multiple | Immunostaining |
| --- | --- | --- | --- | --- | --- | --- | --- | --- | --- | --- | --- | --- | --- | --- | --- |
| 1 | Anthony  et al. (3) | 46 years | M | Head | 1.6 | Pancreaticoduodenectomy | None | - | + | + | + | + | Solid and cystic | Multiple |  |
| 2 |  | 35 years | M | Tail | multiple | Distal pancreatectomy | Epigastric pain | + | + | - | + | + | NR | NR |  |
| 3 |  | 58 years | M | Head | 1 | Autopsy | None | - | + | + | + | + | NR | NR |  |
| 4 | Burt  et al. (4) | 34 weeks | F | Diffuse | 11.5 | Total pancreatectomy | Abdominal pain | - | + | + | + | + | Solid and cystic | Multiple |  |
| 5 | Flaherty  et al. (5) | 20 month | F | Head | 9 | Local resection | Abdominal pain  Distention | - | + | - | + | + | Solid and cystic | Solitary |  |
| 6 | Izbicki  et al. (6) | 25 years | M | Head | 10.6 | Pancreaticoduodenectomy | Epigastric pain | - | + | + | + | + | Solid and cystic | NR |  |
| 7 | Wu  et al. (7) | 39 years | M | Head | 8 | Pancreaticoduodenectomy | Epigastric pain,  Weight loss | + | + | NR | + | + | Solid and cystic | Solitary |  |
| 8 | McFaul  et al. (8) | 29 years | M | Head | 1 | Pancreaticoduodenectomy | Abdominal pain  Vomiting, Weight loss | + | NR | + | + | - | Solid | Solitary |  |
| 9 |  | 62 years | M | Head | 3.5 | Pancreaticoduodenectomy | Abdominal pain  Vomiting, Weight loss | + | NR | + | + | - | Solid | Solitary |  |
| 10 | Pauser  et al. (9) | 36 years | F | Head | 7 | Pancreaticoduodenectomy | Epigastric pain | - | + | - | + | + | Solid and cystic | Solitary | CD34, CD117 |
| 11 |  | 55 years | F | Head | 3 | Distal pancreatectomy | Abdominal pain | - | + | - | + | + | Solid and cystic | Solitary | CD34, CD117 |
| 12 | Pauser  et al. (10) | 51 years | M | Tail | 3 | Local resection | None | - | + | - | + | + | Solid | Solitary | CD34, CD117, CK8, bcr-2 |
| 13 |  | 54 years | F | Body | 2 | Distal pancreatectomy | Abdominal discomfort | - | + | - | + | + | Solid | Solitary | CD34, CD117, CK8, bcr-2 |
| 14 | Nagata  et al. (11) | 58 years | F | Body | 2 | Distal pancreatectomy | None | - | + | - | + | + | Solid | Solitary | CD34, CD117, Ki-67 (Negative for S-100, a-SMA, desmin and bcr-2) |
| 15 | Durczynski  et al. (12) | 69 years | M | Body | 3 | Central pancreatic resection | None | - | + | + | + | + | Solid | Solitary | Chromogranin, S-100, desmin (Negative for CD34 and CD117) |
| 16 | Kersting  et al. (13) | 67 years | M | Head | 5 | Total pancreatectomy | None | - | + | NR | + | + | Solid | Solitary |  |
| 17 | Sueyoshi  et al. (14) | 14 month | M | Tail | 19 | Local resection | Abdominal Distension | - | + | + | + | + | Solid and cystic | Multiple |  |
| 18 | Kim  et al. (15) | 52 years | F | Head | 2.2 | Pancreaticoduodenectomy | Abdominal discomfort | - | + | NR | + | + | Solid and cystic | Solitary | CD34, CD117, synaptophysin (Negative for chromogranin, Ki-67 and desmin) |
| 19 | Sampelean  et al. (16) | 46 years | M | Head | 0.9 | Pancreaticoduodenectomy | Epigastric pain,  Gastric reflex  Weight loss | - | + | NR | + | + | Solid and cystic | Solitary |  |
| 20 | Addeo  et al. (17) | 61 years | F | Body | 2.7 | Distal pancreatectomy | None | - | + | + | NR | + | Solid | Solitary | bcr-2 (negative for CD34 and CD117) |
| 21 | Yamaguchi  et al. (18) | 78 years | F | Head | 1.7 | Resected (details unknown) | None | - | + | - | + | + | Solid | Multiple | CD34, S-100 |
| 22 |  | 61 years | F | Head | 4 | Resected (details unknown) | Abdominal pain | - | + | - | + | + | Solid | Solitary | CD34, S-100 |
| 23 |  | 71 years | F | Diffuse | 5 | Resected (details unknown) | None | - | + | - | + | + | Solid and cystic | Solitary | CD34, CD117, S-100 |
| 24 |  | 66 years | M | Head | 1.5 | Resected (details unknown) | None | - | + | - | + | + | Solid | Solitary | CD34. S-100 |
| 25 |  | 65 years | M | Head | 4 | Resected (details unknown) | Weight loss | - | + | - | + | + | Solid | Solitary | CD34. S-100 |
| 26 |  | 59 years | F | Tail | 1 | Resected (details unknown) | Abdominal pain | - | + | - | + | + | Solid | Solitary | CD34, CD117, S-100 |
| 27 |  | 53 years | M | Head | 8 | Resected (details unknown) | Abdominal pain | - | + | - | + | + | Solid and cystic | Solitary | CD34. S-100 |
| 28 |  | 53 years | M | Head | 2.5 | Resected (details unknown) | None | - | + | - | + | + | Solid and cystic | Solitary | CD34, CD117, S-100 |
| 29 | Inoue  et al. (19) | 65 years | M | Head | 4 | Pancreaticoduodenectomy | Obstructive jaundice | - | + | - | + | + | Solid | Solitary | CD34, S-100 |
| 30 | Present case | 68 years | M | Head | 4 | Pancreaticoduodenectomy | None | - | Decreased | Decreased | Decreased | + | Solid and cystic | Multiple | CK7,19 S100 |

NR: Not recorded
